# The implementation of DRG-based hospital reimbursement in Switzerland: A population-based perspective

**DOI:** 10.1186/1478-4505-8-31

**Published:** 2010-10-16

**Authors:** André Busato, Georg von Below

**Affiliations:** 1 University of Bern Institute for Evaluative Research in Medicine Stauffacherstrasse 78 CH-3014 Bern, Switzerland; 2 Robinsonweg 97 CH-3006 Bern BE, Switzerland

## Abstract

**Background:**

Switzerland introduces a DRG (Diagnosis Related Groups) based system for hospital financing in 2012 in order to increase efficiency and transparency of Swiss health care. DRG-based hospital reimbursement is not simultaneously realized in all Swiss cantons and several cantons already implemented DRG-based financing irrespective of the national agenda, a setting that provides an opportunity to compare the situation in different cantons. Effects of introducing DRGs anticipated for providers and insurers are relatively well known but it remains less clear what effects DRGs will have on served populations. The objective of the study is therefore to analyze differences of volume and major quality indicators of care between areas with or without DRG-based hospital reimbursement from a population based perspective.

**Methods:**

Small area analysis of all hospitalizations in acute care hospitals and of all consultations reimbursed by mandatory basic health insurance for physicians in own practice during 2003-2007.

**Results:**

The results show fewer hospitalizations and a relocation of resources to outpatient care in areas with DRG reimbursement. Overall burden of disease expressed as per capita DRG cost weights was almost identical between the two types of hospital reimbursement and no distinct temporal differences were detected in this respect. But the results show considerably higher 90-day rehospitalization rates in DRG areas.

**Conclusion:**

The study provides evidence of both desired and harmful effects related to the implementation of DRGs. Systematic monitoring of outcomes and quality of care are therefore essential elements to maintain in the Swiss health system after DRG's are implemented on a nationwide basis in 2012.

## Introduction

In 2007, the Swiss Parliament passed the new hospital financing law which includes a DRG-based tariff structure (Diagnosis Related Groups) to be introduced nationwide by 2012. An organizational framework was created (SwissDRG AG) and was mandated by the Swiss federal government to develop and implement a DRG-based system for hospital financing. The activities of SwissDRG were mainly focused on technical issues related to the adaptation of classification algorithms and the definition of cost weights suitable for the Swiss health system. In parallel, the discharge dataset of all hospitalization in Swiss hospitals maintained by the Swiss Federal Statistical Office (Medizinische Statistik der Krankenhäuser) was modified and includes in its most recent version all necessary data for classification of diagnoses and procedures and DRG-grouping using the SwissDRG grouper. The SwissDRG classification and grouper is based on the German G-DRG system[1-3]. Additional projects were initiated in order to provide the scientific basis of DRG-based hospital reimbursement mostly from an economic perspective[4]. The respective effects of introducing DRGs anticipated for providers and insurers are therefore relatively well known but it remains less clear what effects DRGs will have on served populations.

DRG-based hospital reimbursement is not simultaneously realized in all cantons and several cantons already implemented DRG-based financing schemes irrespective of the national agenda based on 3M's AP-DRG system[5]. A setting that provides an unique opportunity to analyze the respective effects within a single health system. We designed therefore a population based study relying on utilization based health service areas. The purpose is to quantify DRG related effects on the following indicators:

- Hospitalization rates

- Duration of hospitalizations

- In-hospital mortality

- 90-day rehospitalization rates

- Resource utilization of in- and outpatient care

- Relocation of resources from inpatient to outpatient care

## Methods

The study is based on the complete dataset of all hospital discharges in Switzerland for the years 2003-2007 (Swiss Federal Statistical Office) and on the complete claims data at the expense of basic health insurance of physicians in own practice for the same period. Claims data at the physicians level were obtained from santésuisse, the umbrella organization of all Swiss health insurers. Discharge records were used to construct 86 Hospital service areas (HSA) by applying methods described in earlier publications[6,7]. Discharge records of patients in psychiatry and rehabilitation institutions were excluded from these procedures as these data could not be grouped into DRG's on a reliable basis. Discharges of hosptialisations in an ambulatory or semi-ambulatory setting were also excluded from the analysis. HSAs of acute care hospitals were therefore the units of observation of our study and allowed an analysis of the data at a small, utilization based, geographic scale. The methodological approach of using health service areas has the advantage that it directly reflects where patients actually receive care irrespective of cantons or other administrative borders [6-8]. This type of approach is well established and has been an indispensable source of information within the current efforts to reform US healthcare[9].

To ensure higher levels of representativeness the minimum localization index was set to 40%, which implies that 40% of the residents of an area were treated by a hospital within their own region of residency. Health service areas were classified into DRG or non-DRG areas depending on hospital location in cantons with the respective reimbursement systems in place.

Socio-demographic and mortality data at the community level were obtained from the Swiss Federal Statistical Office (Statweb/Superweb) and were aggregated with claims data of ambulatory care at the level of hospital service areas. We used unadjusted hospitalization and mortality rates as there are currently no valid age distributions at the community level available. Duration of hospitalizations, in-hospital mortality per 1000 hospitalizations, 90-day rehospitalization rates and DRG cost weights were calculated using the respective variables available in the discharge records[1]. Referrals between hospitals (administrative discharges) and hospitalizations coded as "referral within 24 hours" were excluded from calculations of 90-day rehospitalization rates. DRG cost weights accounting for outliers of length of stay were calculated according to version 6.0 of the specifications of DRG-Suisse[2]. TARMED reimbursement tariff values were used as cost weights of the regional utilization of services provided by ambulatory physicians in own practices. This TARMED-scheme was introduced in 2004 on a national level. It includes a system to steer cost development based on a reimbursement factor for medical services negotiated annually between medical associations, health insurers and health authorities on a cantonal level[10]. TARMED cost weights were calculated by dividing claims in Swiss Francs by these annual reimbursement factors for each canton and year. Per capita utilization of both in- and outpatient services were calculated based on these cost weights for each HSA and the ratio of the regional sums of DRG vs. TARMED cost weights was used to quantify the relocation of treatment volume between inpatient and outpatient care across HSAs and time.

## Results

Small area procedures yielded 86 health service areas and on average 55% of the population were hospitalized within their own area of residence and 87% within their own canton. One canton implemented DRG hospital reimbursement before 2003 (Waadt 2002) and nine other cantons changed to DRG reimbursement during the study period[11]. The number of health service areas with DRG reimbursement increased therefore from 8 in 2003 (8.3% of hospitalizations) to 26 in 2007 (29.8% of hospitalizations). For 2003-2007 the number of hospitalizations increased by 8.7%, ambulatory consultations by 7.3% and mortality decreased by 3%. Total sums of hospitalizations and consultations in ambulatory practices for 2003-2007 and the respective distribution of HSAs across the two reimbursements systems are given in table 1.

**Table 1:** Sums of hospitalizations and consultations in ambulatory practices for 2003-2007 across type of hospital reimbursement

Year	2003	2004	2005	2006	2007
Nr of HSAs	86	86	86	86	86
without DRG	78	74	66	61	60
with DRG	8	12	20	25	26

# Hospitalizations	1022503	1048582	1054388	1080697	1111455
without DRG	937364 (91.7)^a^	924669 (88.2)	827957 (78.5)	819227 (75.81)	779978 (70.2)
with DRG	85139 (8.3)	123913 (11.8)	226431 (21.5)	261470 (24.19)	331477 (29.8)

# Consultations in ambulatory practices	37416811	38660566	39990535	39222161	40164883
without DRG	34465115 (92.1)^a^	34430545 (89.1)	32154592 (80.4)	30567734 (77.9)	28639951 (71.3)
with DRG	2951696 (7.9)	4230021 (10.9)	7835943 (19.6)	8654427 (22.1)	11524932 (28.7)

^a ^proportion in % within year and type of care

Differences and temporal variation of hospitalization rates, mortality and per capita cost weight volume across the two reimbursement systems are given in table 2. The data show lower hospitalization rates (-2.8%), shorter hospital stays (-10%), higher 90-day rehospitalization rates (+13.5%), reduced in-hospital mortality (-3.1%), almost equal inpatient cost weights per 1000 population (-1.1%) and lower outpatient cost weights (-1.3%) in areas with DRG reimbursement.

**Table 2:** Averages of 86 hospital service areas across type of hospital reimbursement

	**2003 (78/8)**^**a**^	2004 (74/12)	2005 (66/20)	2006 (61/25)	2007 (60/26)	% difference 2003(04)-2007	Mean 2003(04)-2007	**% Difference**^**b**^
Hospitalization rate/1000 inhabitants	143.58	145.85	145.97	147.87	150.29	4.67	146.71	
without DRG	144.55	146.81	146.25	149.23	152.22	5.31	147.57	
with DRG	134.18	139.94	145.05	144.56	145.82	8.67	143.51	-2.76

Duration of hospital stay (days)	8.76	8.28	8.14	7.87	7.60	-13.28	8.13	
without DRG	8.91	8.48	8.29	7.99	7.63	-14.31	8.30	
with DRG	7.34	7.03	7.64	7.56	7.52	2.48	7.48	-9.97

In-hospital mortality^c^	22.09	20.88	21.02	20.38	20.66	-6.47	21.01	
without DRG	22.18	21.27	21.15	20.09	20.71	-6.66	21.14	
with DRG	21.19	18.51	20.61	21.08	20.56	-2.95	20.50	-3.05

Mortality^d^	8.61	8.29	8.36	8.18	8.35	-3.10	8.36	
without DRG	8.62	8.37	8.49	8.21	8.50	-1.41	8.45	
with DRG	8.56	7.74	7.92	8.09	8.00	-6.57	8.02	-5.03

3 month rehospitalization rate	0.15	0.18	0.18	0.18	0.19	23.00	0.18	
without DRG	0.15	0.18	0.18	0.18	0.18	21.64	0.17	
with DRG	0.17	0.19	0.21	0.19	0.20	16.91	0.19	13.45

DRG cost weights per 1000 residents	144.56	141.39	146.25	150.02	151.85	5.04	146.81	
without DRG	144.92	140.69	146.89	151.88	153.46	5.89	147.14	
with DRG	141.06	145.75	144.11	145.46	148.12	5.01	145.58	-1.07

TARMED tariff values per 1000 residents	-^e^	594.33	625.14	621.81	643.20	8.22	621.12	
without DRG	-	597.62	626.66	629.79	643.52	7.68	623.03	
with DRG	-	574.06	620.15	602.34	642.44	11.91	615.11	-1.27

Ratio DRG vs. TARMED cost weights	-	0.24	0.24	0.25	0.24	-0.54	0.24	
without DRG	-	0.24	0.24	0.25	0.25	2.00	0.24	
with DRG	-	0.26	0.23	0.24	0.23	-9.99	0.24	-0.84

^a ^ratio of number of non-DRG areas vs. DRG areas

^b ^difference of means for 2003-2007 between reimbursement systems in %

^c ^# deaths/1000 hospitalizations

^d ^# deaths/1000 residents

^e ^TARMED was introduced in 2004

**Figure 1:**
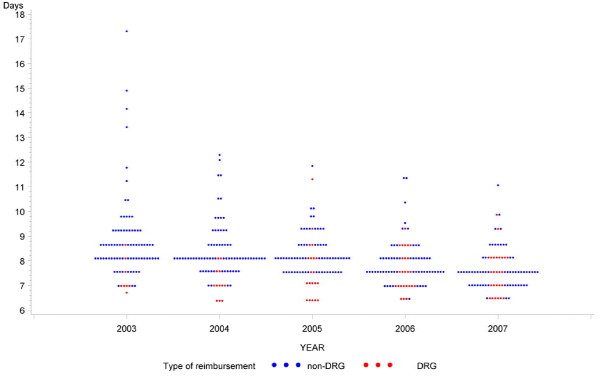
**Average length of stay 2003-2007 (86 health service areas)**. Each dot represents a health service area of a given year

Temporal variation indicates considerable differences for some of these variables. Hospitalization rates in non-DRG areas increased by 5.3% vs. 8.7% in DRG areas. A marked drop of 1.3 days (-14.3%) of less length of stay is seen in non-DRG areas whereas length of stay increased by 0.2 days (+2.5%) in DRG areas. Figure 1 visualizes these patterns and also indicates a decrease of geographic variation of length of stay over time.

In-hospital mortality decreased by 6.7% in non-DRG areas compared 3.0% in DRG areas. Overall mortality was 5% lower in DRG areas and between 2003-2007 mortality fell by 6.6% in DRG areas vs. 1.4% in non-DRG areas. Temporal change of 90-day rehospitalization rates indicates a 21.6% increase in non-APDRG areas vs. a 16.9% increase in DRG areas. Some temporal differences between the two reimbursement systems have to be noted. DRG cost weights increased by 5.9% in non-DRG areas vs. 5.0% in DRG areas and TARMED data of physicians in own practices indicate a 7.7% increase of outpatient cost weights for 2004-2007 in non-DRG areas vs. 11.9% in DRG areas. The average ratio between the two cost weights for 2004-2007 indicates almost equal ratios in DRG or non-DRG areas (-0.8 difference) however, considerable temporal differences between the two forms of reimbursement are apparent. In DRG areas the relationship between in- and out-patient care increased by 10% in favor of outpatient care compared to a 2% decrease in non-DRG areas.

## Discussion

The study documents effects related to the stepwise implementation of DRG-based hospital reimbursement within the current reform of Swiss health care aimed at stabilizing overall expenditures and at improving transparency and quality of care. The results are consistent with the anticipated reduction of length of stay and fewer hospitalizations. The results also show an almost equal burden of cost weights per population irrespective of type of reimbursement but 90-day rehospitalization rates were considerably higher in areas where DRG-based hospital reimbursement is already in place. A relocation of resources from in- to outpatient care of practice-based physicians after implementation of DRGs was also observed in the analysis.

### Hospitalization rates, per capita cost weights and mortality

The data indicate fewer hospitalizations in DRG areas and a different temporal development compared to non-DRG areas. However differences were relatively small and patterns of per capita DRG cost were almost identical between the two types of hospital reimbursement.

Several studies indicate an association between severity of disease and coding practices after the implementation of DRG-based reimbursement systems [12-14]. Our data do not support such an association as they provide no indication of a considerable discrepancy between regional levels of burden of disease as expressed by inpatient cost weights and rates of in-hospital mortality and overall mortality respectively. Some differences between in-hospital and overall mortality have to be noted in this context. In-hospital mortality is not only a function of inpatient health but also related to availability and utilization of care institutions for terminally ill patients. The location of death may consequently differ between reimbursement systems [15]. We consider therefore overall mortality as a better indicator of health status and medical needs of populations.

### Length of stay

Numerous studies performed in various countries document a reduction of length of stay after implementing DRG-based reimbursement systems[12,13]. Our data show that reduced length of stay is not necessarily related to the introduction of DRGs as length of stay in non-DRG areas was reduced by 14% during the five year study period and reached almost the same value as observed in DRG areas in 2007. Length of stay reached therefore levels of other comparable OECD countries[16], irrespective of type of hospital reimbursement. Overall economic pressure on health care may be a more important factor in this context. Concerns raised by the National Advisory Commission on Biomedical Ethics[17] about damaging effects of shorter hospitalizations and consequent poorer quality of care may therefore be obsolete with reference to DRGs as a causal factor.

### Rehospitalization rates

Rehospitalization rates are considered important quality indicators of inpatient care as potentially avoidable readmissions can be the consequence of an adverse event or a too early discharge of a prior hospitalization[18,19]. Alternatively, DRG based remuneration can cause changes in case organization as simultaneously occurring medical problems are treated as separate cases (case splitting)[20]. Our data show consistent higher three-month rehospitalization rates in DRG areas for the entire study period and eventually imply suboptimal quality and potentially poor organization of care in these areas. The data also show that rehospitalization rates are not necessarily inversely related with length of stay which remained almost stable in DRG areas but declined considerably in non-DRG areas. Our findings are therefore consistent with other research indicating that high rehospitalization rates are not an inevitable price of early discharges [18]. However, condition unspecific rehospitalization data as used in this study have only limited value and a population based study cannot provide in-depth insight into treatment episodes of individual patients within different settings of hospital reimbursement. Our data can therefore only emphasize the fact that further condition specific research is needed. Research efforts should not only be aimed at the effect of short hospital stays but also at other damaging changes of the care process including fragmentation and depersonalization anticipated with the implementation of DRG-based hospital reimbursement[17].

### Shift of cost weights to outpatient care

The use of cost weights in this study allows a direct representation of treatment volume irrespective of attached monetary values reimbursed by health insurance. Our results reflect therefore the effective use of resources at a small area scale unbiased by differing reimbursement factors between cantons and across time.

The overall difference for 2003-2007 of the cost weight ratio of inpatient vs. outpatient care indicates no major difference between reimbursement systems. However in DRG areas considerable reallocation to outpatient setting occurred and a systematic cooperation between hospitals and practice-based physicians (integration of care pathways) could be achieved. This interpretation is, however, compromised as our study provides no data of treatment volume in outpatient departments of hospitals. The effective extent of reallocation is therefore likely to be higher as hospitals may have moved care within their own facilities.

### Strengths and limitations

Our study is based on the complete data of all hospitalizations in acute care hospitals and all consultations reimbursed by mandatory basic health insurance for physicians in own practice. The results are therefore fully representative for these two settings of care for 2003-2007. However, data of ambulatory hospital departments were not available because of concerns of santésuisse about confidentiality of the data. The respective volume of care and its interaction with both inpatient and non-hospital outpatient care are therefore unknown. Similar limitations have also to be noted with reference to the lack of data for psychiatry and rehabilitation. A further restriction is our reliance on data of mandatory health insurance for the outpatient setting which does not include out of pocket payments and services related to accidents or invalidity which are both included in the inpatient data.

This study was performed with a population based perspective in order to identify regional differences in the per capita utilization of health related resources as a function of type of hospital reimbursement. This is in contrast with most other research in this domain that is usually performed with a provider or hospital based perspective. We therefore have to acknowledge some limitations in this context. The spatial model of health services areas with an average proportion of 55% local hospitalizations is not perfect. However, 87% of patients were hospitalized within their own canton of residence and since DRGs were introduced mostly on a cantonal level we consider our spatial model as a reasonable trade-off between the requirements of high geographic resolution and a valid representation of the regional utilization of resources. Another limitation refers to the fact that utilization based health service areas are usually not congruent with administrative boundaries and there is consequently a lack of demographic and socio-economic data at this level that would allow an analysis of the related effects on resource utilisation.

## Conclusions

The study provides empirical evidence of both desired and harmful effects related to the implementation of DRG-based hospital reimbursement within the Swiss health system. There is a desired shift to practice-based outpatient care in the sense of a systematic cooperation between hospitals and physicians in own practice. Harmful effects, related to higher rehospitalization rates question the value of a DRG-based payment system from a quality of care and from an economic perspective. The analysis provided no other evidence of major negative consequences of converting the current system of hospital reimbursement into a DRG-based system. Systematic monitoring of outcomes and quality of care remains nevertheless an essential element to be maintained in the Swiss health system after implementation of DRGs on a nationwide basis.

## Competing interests

The authors declare that they have no competing interests.

## Authors' contributions

AB obtained the data, performed the statistical analysis and wrote a first draft of the manuscript. GvB reviewed the manuscript and provided considerable input with reference to DRG-based hospital reimbursement within the Swiss health system. Both authors read and approved the final manuscript.
